# Retrospective Observational Study of Daytime Add-On Administration of Zopiclone to Difficult-to-Treat Psychiatric Inpatients With Unpredictable Aggressive Behavior, With or Without EEG Dysrhythmia

**DOI:** 10.3389/fpsyt.2021.693788

**Published:** 2021-08-17

**Authors:** Alfonso Ceccherini-Nelli, Elena Bucuci, Lisa Burback, Daniel Li, Maryam Alikouzehgaran, Zahid Latif, Kevin Morin, Karthikeyan Ganapathy, Manhaz Salsali, Ubaid Abdullah, Wanda Westwood, Janice Orris, Patrick J. White

**Affiliations:** ^1^Department of Psychiatry, University of Alberta, Edmonton, AB, Canada; ^2^Alberta Health Care Services, Edmonton, AB, Canada

**Keywords:** violence, zopiclone, GABA, neurocognitive disorders, neurodevelopmental disorders, schizophrenia, difficult-to-treat, inpatients

## Abstract

Managing violent behavior is a particularly challenging aspect of hospital psychiatric care. Available pharmacological interventions are often unsatisfactory.

**Aim:** To assess the effectiveness and safety of daytime zopiclone add-on administration in violent and difficult-to-treat psychiatric inpatients.

**Methods:** Chart review of inpatients treated with daytime zopiclone, between 2014 and 2018, with up to 12 weeks follow-up. Effectiveness was retrospectively assessed with the Clinical Global Impression rating scale (CGI) and the frequency and severity of aggressive incidents recorded with the Staff Observation Aggression Scale-Revised (SOAS-R).

**Results:** Forty-five (30 male, 15 female) cases, 18–69 years age range, average (SD) baseline CGI-S score of 5.4 (1.0), and a variety of diagnoses. Sixty-nine percent showed CGI-S improvement of any degree. For patients with at least one aggressive incident within 7 days prior to initiation of zopiclone (*N* = 22), average (SD) SOAS-R-Severity LOCF to baseline change was −3.5 (2.7) *P* < 0.0001. Most patients reported no side effects; 24% reported one or more side effects, and 11% discontinued zopiclone due to sedation (4), insomnia (1) or slurred speech (1). No SAEs were recorded. Zopiclone maximum daily dose correlated with CGI-S baseline-to-LOCF change (rho = −0.5, *P* = 0.0003). The ROC AUC of zopiclone maximum daily dose and improvement on CGI-S was 0.84 (95% CI 0.70–0.93, *P* < 0.0001). The ROC AUC of zopiclone maximum daily dose and SOAS-R-N improvement was 0.80 (95% CI 0.58–0.92; *P* = 0.0008) and maximum Youden's index value was achieved at a dose of >30 mg.

**Conclusions:** Zopiclone doses >30 mg daily achieved the best anti-aggressive effect.

## Introduction

Zopiclone is a short-acting hypnotic agent with a pharmacological profile similar to benzodiazepines. Zopiclone pharmacological properties are hypnotic, sedative, anxiolytic, anticonvulsant, and muscle relaxant. These effects are related to a specific positive allosteric modulation (PAM) of central receptors belonging to the GABAA macromolecular complex, modulating the chloride ion channel's opening (1).

Zopiclone is often found in the blood of those with drug abuse, self-harm, overdose, and road accidents. It is overused in the elderly, and many adverse consequences, including injury, have been associated with it (2). Zopiclone prescription and use should have the same concerns as those for benzodiazepine pharmacotherapy (2).

Nonetheless, in 2014 we made a serendipitous observation of remarkable clinical effects after daytime administration of zopiclone in a treatment-resistant patient with a Major Vascular Neurocognitive Disorder.

This patient (listed as case 41 in the Supplementary Table A) was a man in his 60s, admitted to a psychiatric intensive care unit due to episodes of severe agitation and homicidal violence. He had suffered cardiac arrest and severe hypoxic brain injury at the age of 47. On numerous occasions, he became violent with staff on the unit. One of his behaviors was to insist that he still had a wife and a home to return to or that he had to drive his truck to work. He was frequently mute, except for occasional short incomplete sentences. He showed mild ataxic cerebral palsy. Multiple psychotropic drugs, including benzodiazepines, had been poorly tolerated or ineffective in controlling his recurrent aggressive outbursts.

A recent resting-state electroencephalogram (EEG) showed intermittent brief runs of high amplitude 1–3 Hz waves predominantly over the right side leads and frontally and frequent 4–8 Hz waves over the central and temporal regions. Considering a previous report (3) of patients in minimally conscious states and EEG with excess expression of slow rhythms becoming alert and regaining motor function after zolpidem administration (pharmacologically similar to zopiclone), we cautiously administered zopiclone during daytime in this patient, up to a combined daily dose of 22.5 mg. The results were remarkable. He regained interest in the environment and recreational activities. His gait improved, and he regained control of fine movements of his hands. The frequency and complexity of his speech improved. Most importantly, there was a complete resolution of any aggressive and exit-seeking behavior for the remaining 5 weeks of his hospitalization. While receiving daytime doses of zopiclone, a new resting-state EEG was normal with no evidence of intrusion of high amplitude delta and theta waves.

Since this first case, several physicians in our hospital have tried adjunctive daytime administration of zopiclone for complex psychiatric patients, who either did not respond or could not tolerate conventional treatments, especially if the patient had an abnormal baseline EEG with excess theta/delta expression. This started with using zopiclone for difficult to treat agitation, aggression, or severe impulsive behavioral problems. However, because of observed clinical improvements in mood, cognition and motricity in some patients, it was also tried for other complex psychiatric presentations, where aggression was not the primary problem. This report summarizes a chart review of all inpatients treated with daytime zopiclone between 2014 and 2018. The analysis focused on assessing clinical global improvement, improvement of aggressive behavior, safety, and potential association of outcomes with demographic and clinical variables, including baseline EEG findings and zopiclone total daily dose.

## Methods

### Clinical Setting

All patients analyzed in this study were inpatients of Alberta Hospital Edmonton (AHE) General Adult Program. AHE is the largest psychiatric hospital in the Canadian province of Alberta. The general psychiatric adult section of the hospital comprises three secure general adult acute units (84 beds) and one secure psychiatric intensive care unit (16 beds) (PICU). The PICU at AHE is the largest in the province and accepts patients directly from all Emergency Departments and acute general psychiatric units in the Edmonton Zone (~1.4 million population). The PICU also accepts transfers from other adult PICUs, and from forensic wards of patients released from any legal hold still requiring PICU level of care. In addition, the PICU receives patients from other services including intellectual disability, rehabilitation, and old age inpatient programs. Consequently, the PICU at AHE is a tertiary multidisciplinary acute inpatient unit, specialized in the care of highly complex and frequently treatment resistant patients posing a high risk of harm to self or others. Most patients described in this paper were initiated on zopiclone while they received care in the PICU.

### Data Collection

Using our hospital pharmacy database, we identified 65 inpatients who had been prescribed daytime zopiclone between 2014 and 2018 by 12 different physicians. From this list, we excluded: nine patients who had not been adherent to treatment for at least three continuous days; one patient who was tried on zopiclone to improve essential tremor and not for treatment of psychiatric symptoms; finally, we were unable to include 10 patients because their clinical records could not be made available before a set deadline. Therefore, the clinical records of 45 patients were reviewed to extract a narrative summary of the cases, with attention to any reported side effects. For all patients excluded from this retrospective review, any available information about untoward events was included in our safety analysis. The recorded diagnoses (primary, secondary and differential) were those proposed by the admitting physician and were never changed by the raters. Retrospectively we rated mental state changes using the Clinical Global Impression rating scale (CGI) (4) and the Staff Observation Aggression Scale-Revised (SOAS-R) (5). The CGI includes three subscales: Severity scale (CGI-S), Improvement scale (CGI-I) and Efficacy Index (CGI-EI). The SOAS-R includes one Visual Analog Scale of Severity (VASS) of the aggressive incident and five subscales. We report only the number of aggressive incidents per week (SOAS-R-N) and their severity (SOAS-R-S) on a scale from 0 to 10, extrapolated from the VASS. Weekly CGI and SOAS-R scores were rated for up to 20 weeks, a minimum of 1 to maximum 8 weeks before zopiclone initiation and up to 12 weeks or until time of discharge (if sooner), while treated with daytime zopiclone. For patients who remained in hospital more than 12 weeks after commencing zopiclone, we reviewed their charts to collect information about adverse events eventually occurred until discharge, without rating either the CGI or SOAS-R beyond the first 12 weeks.

The list of patients was rearranged by name alphabetical order, AC-N reviewed charts starting from the top and EB from the bottom of the list. Approximately an equal number of charts were reviewed by AC-N and EB. At the beginning of the review AC-N and EB reviewed three charts together to discuss and agree how to rate outcomes using the study instruments. Both physician and nursing notes were reviewed.

### Relationship of Demographic and Clinical Variables to Outcomes

We analyzed the relationship of gender, age, diagnosis, chronicity indicators, presence/absence of EEG excess delta/theta rhythm, severity, and frequency of aggressive incidents before starting the treatment, zopiclone treatment duration and dose to score changes of CGI subscales and the frequency and severity of aggressive incidents as recorded on the SOAS-R.

### Statistical Analysis

Descriptive statistics were used to quantify demographic and clinical variables. Differences in clinical variable scores before and after treatment with zopiclone were assessed with a non-parametric Mann-Whitney test. The sample was stratified according to different criteria, and efficacy was compared among these groups. When the stratification criterion was dichotomous, the resulting groups could be compared with the Mann-Whitney test. When the stratification criterion was a continuous variable, the association between this criterion and the outcome variable was quantified with Spearman's coefficient of rank correlation (rho). Because of multiple comparisons tested in this study (*N* = 10), the alpha value 0.05 was corrected to 0.005, using Bonferroni's method. A receiver operating characteristic curve (ROC) was used to determine the optimal dose of zopiclone to improve the outcome measures. ROC is a statistical tool to assess the accuracy of a test. The ROC curve is widely accepted to select an optimal cut-off point and compare diagnostic tests' accuracy. It is a plot of the true-positive rate against the false-positive rate for different cut-off points. Other applications of the ROC curve for various situations in clinical trials and drug development have been proposed (6). In this paper we used MedCalc^®^ Statistical Software (MedCalc Software Ltd, Ostend, Belgium; https://www.medcalc.org; 2021).

## Results

### Demographic and Clinical Characteristics

As shown in Table 1, this cohort comprised 45 psychiatric inpatients, of whom 30 (66.6%) were men. The average (SD) age was 38.4 (14.6) years, and the estimated age of the first episode of illness was 23.7 (13.7) years. The primary diagnosis recorded at the time of hospital admission was: schizophrenia and other psychotic disorders (20 patients, 44%), of whom 13 were schizoaffective; neurodevelopmental disorders (8, 18%) of whom 3 were autism spectrum; neurocognitive disorders (5, 11%) of whom 2 were vascular; bipolar and related disorders (5, 11%); borderline or antisocial personality disorders (3, 7%); depressive disorders (2, 4%); substance-related disorders (2, 4%).

**Table 1:** Patients' characteristics.

Total, No	45
Sex	Male 30, Female 15
Age, years - Median, average (SD), range	34, 38.4 (14.6), 18–69
Principal Diagnosis	20 Schizophrenia Spectrum and Other Psychotic Disorders (Schizoaffective 13)
	8 Neurodevelopmental disorders (Autism Spectrum 3)
	5 Neurocognitive disorders (vascular 2)
	5 Bipolar and related Disorders
	3 Personality disorders (Borderline and/or antisocial)
	2 Substance-Related Disorders
	2 Depressive Disorders
Age of 1st episode of illness, years—Median, average (SD), range	23, 23.7 (13.7), 2–56
Duration of illness, years—Median, average (SD), range	15, 21.1 (20.5), 0–69
Current hospitalization length of stay (LOS), days—Median, average (SD), range	147, 256.3 (322.9), 13–1,435
Resting EEG Available (Yes)	34
EEG excess delta/theta rhythm (yes)	18
Zopiclone treatment duration, until discharge or discontinuation, days—Median, average (SD), range	37, 84.4 (113.7), 3–498
Zopiclone maximum daily dose, mg—Median, average (SD), range	30, 38.0 (18.9), 5–60
Baseline CGI-S—Median, average (SD), range	6, 5.4 (1.0), 3–7
CGI-S LOCF—Median, average (SD), range	4, 4.0 (1.26), 3–7
CGI-S any Improvement, *n*. (%)	31 (69)
CGI-S ≥ 2 score Improvement, *n*. (%)	19 (42)
CGI-S LOCF—Baseline change—Median, average (SD), range	−1, −1.5 (1.4)^*^, −5 to +1
CGI-I LOCF—Median, average (SD), range	2, 2.5 (1.3), 1–5
CGI-I <3, *n*. (%)	27 (60)
CGI-EI LOCF—Median, average (SD), range	5, 6.2 (4.7), 1–14
SOAS-R-N average pre-zopiclone—Median, average (SD), range	0.3, 0.6 (0.8), 0–3
SOAS-R-N last week pre-zopiclone—Median, average (SD), range	0, 0.9 (1.2), 0–6
SOAS-R-N average post-zopiclone—Median, average (SD), range	0, 0.2 (0.3), 0–1.3
SOAS-R-N LOCF—Median, average (SD), range	0, 0.1 (0.2), 0–1
SOAS-R-N post-pre-Z change—Median, average (SD), range	−0.2, −0.4 (0.7)^+^, −2.8 to 0.2
SOAS-R-N post-pre-Z change only pts with SOAS-R-N ≥ 1 last week pre-zopiclone (*N* = 22)—Median, average (SD), range	−0.5, −08 (0.8)^#^, −2.8 to 0.2
SOAS-R-N LOCF-pre-Z change—Median, average (SD), range	−0.2, −0.6 (0.8)^*^, −3 to 0.9
SOAS-R-S pre-Z average—Median, average (SD), range	1.7, 2.2 (2.4), 8.5–0
SOAS-R-S Post-Z average—Median, average (SD), range	0, 0.7 (0.9), 0–3.8
SOAS-R-S LOCF—Median, average (SD), range	0, 0.3 (1.0), 0–4.5
SOAS-R-S post-pre change—Median, average (SD), range	−0.9, −1.5 (1.8)^∧^, −6.7 to 0.8
SOAS-R-S post-pre change, only pts with SOAS *N* ≥ 1 last week pre-zopiclone (*N* = 22)—Median, average (SD), range	−2.5, −2.5 (1.7)^*^, −6.7 to −0.2
SOAS-R-S LOCF-pre change—Median, average (SD), range	−1.2, −1.9 (2.7)^*^, −8.5 to 3.7
SOAS-R-S LOCF-pre change, only pts with SOAS *N* > 1 last week pre-zopiclone (*N* = 22)—Median, average (SD), range	−3.2, −3.5 (2.7)^*^, −8.5 to 2.0

*CGI-S, Clinical Global Impression-Severity; CGI-I, Clinical Global-Improvement; CGI-EI, Clinical Global Impression-Efficacy Index; LOCF, last observation carried forward*.

*Mann-Whitney test (independent samples) Two-tailed probability:*

* *P < 0.0001*,

+ *P = 0.0094*,

# *P = 0.0002*,

∧ *P = 0.002*.

For comparison, the number and % of diagnostic groups for patients discharged from the acute units of Alberta Hospital Edmonton during the 2 year period 2018-2019^1^ were: schizophrenia and other psychotic disorders (443, 40%); neurodevelopmental disorders (16, 1%); neurocognitive disorders (6, 1%); bipolar and related disorders (174, 16%); personality disorders (43, 4%); depressive disorders (161, 14%); substance-related disorders (182, 16%); anxiety and trauma/stressor-related disorders (87, 8%); chi-square test = 118.6, *P* < 0.0001. It should be noted that some patients with neurodevelopmental or neurocognitive disorders may be transferred temporarily to the PICU and then return to their units, which are not part of the acute general adult program.

A baseline, pre-treatment resting-state EEG was available for 34 patients, of which 18 were abnormal with excess delta/theta slow rhythms.

Median and average (SD) baseline CGI-S scores were 6 and 5.4 (1.0), where a CGI-S score of 6 indicates “severely ill,” and 5 means “markedly ill.” Median, average (SD) and range of current hospital length of stay (LOS) were 147, 256.3 (322.9), 13–1,435 days. The average (SD) duration of illness was 21.1 (20.5) years.

Patients were treated a minimum of 3 consecutive days to a maximum of 498 days, with an average (SD) of 84.4 (113.7) days. The number of zopiclone administrations varied from twice daily to four times daily, with maximum daily dose ranging between 5 and 60 mg, with an average (SD) of 38.0 (18.9).

Average (SD) CGI-S last observation carried forward (LOCF) was 4.0 (1.3), with 31 (69%) of patients showing a reduction of at least 1 point on the CGI-S and 19 (42%) showing a decrease of at least 2 points on the CGI-S. The average (SD) CGI-S change from baseline to LOCF was −1.5 (1.4) (*P* < 0.0001).

Average (SD) CGI-I LOCF was 2.5 (1.3), and the number of patients with CGI-I <3 (much or very much improved) were 27 (60%).

Average (SD) CGI-EI LOCF was 6.2 (4.7), where 6 stands for “decided improvement with side effects that do not significantly interfere with patient's functioning.”

Baseline average (SD) weekly number of aggression incidents (SOAS-R-N) changed from 0.6 (0.8) to 0.2 (0.3) (averaging all weeks) or 0.1 (0.2) (LOCF), after zopiclone administration. Both differences of SOAS-N from baseline were statistically significant (Table 1). SOAS-R-N score change was also statistically significant in the subgroup of patients showing aggressive behavior at baseline (22 patients). Of this subgroup, 20 (91%) patients had a reduced number of aggression incidents from baseline.

Baseline average (SD) severity of aggression incidents (SOAS-R-S) changed from 2.2 (2.4) to 0.7 (0.9) (averaging all weeks) or 0.3 (1.0) (LOCF) after zopiclone administration. Both differences of SOAS-R-S from baseline were statistically significant (Table 1).

None of the patients, who received zopiclone for more than 12 weeks while still in hospital, showed a marked exacerbation of symptoms after the 12th week of observation.

### Safety and Tolerability

No serious adverse events were reported in any of the 45 patients described here, as well as in the other 20 patients known to have been treated with daytime zopiclone. 11 patients (24%) reported one or more side effects; of these, 5 (11%) discontinued zopiclone. The reported side effects were drowsiness and/or sedation (8), tiredness (3), insomnia (2), slurred speech (1). Side effects leading to discontinuation of zopiclone were drowsiness or sedation (4), insomnia (1), slurred speech (1). All reported side effects were temporary or resolved after discontinuation. There was no statistically significant correlation between zopiclone dose and the onset of side effects.

### Relationship of Demographic and Clinical Variables to Outcomes

#### Gender and Age

The average (SD) CGI-S change from baseline to LOCF was −1.5 (1.4) for males and −1.3 (1.8) for females (NS). The average (SD) number of aggression incidents change from baseline to LOCF was −0.6 (0.9) for males and −0.5 (0.8) for females (NS). The average (SD) severity of aggression incidents from baseline to LOCF was −2.3 (2.6) for males and −1.6 (2.5) for females (NS).

Spearman's rho of age and CGI-S change from baseline to LOCF was 0.1 (NS), rho of age and SOAS-R-N change was 0.2 (NS), rho of age and SOAS-R-N change was 0.2 (NS), rho of age and SOAS-R-S change was 0.3 (*P* = 0.046, that is NS after Bonferroni correction).

#### Diagnosis

The average (SD) CGI-S change from baseline to LOCF in the different diagnostic subgroups were −1.3 (1.6) in Schizophrenia spectrum disorders, −1.9 (1.3) in neurodevelopmental disorders, −1.7 (1.3) in neurocognitive disorders, −1.7 (2.1) in bipolar disorders. None of the group differences were statistically significant. The differences in SOAS-R score change between diagnostic subgroups were also not statistically significant.

#### Chronicity Indicators

Illness duration was associated with a lower response to zopiclone, as shown by Spearman's rho of Illness Duration and CGI-S change from baseline to LOCF equal to 0.3 (*P* = 0.0392, NS after Bonferroni correction) (Supplementary Figure 1).

Current hospital length of stay (LOS) was associated with a lower response to zopiclone, as shown by Spearman's rho of LOS and CGI-S change from baseline to LOCF equal to 0.4 (*P* = 0.0044, significant after Bonferroni correction).

#### Baseline Resting EEG With Excess Delta/Theta Rhythms

An improvement of CGI-S was observed in 13/18 patients with excess slow rhythms EEG and 12/16 patients with normal EEG (Odds ratio = 0.9, NS).

In patients with aggressive behavior in the 7 days prior to zopiclone initiation, a reduction of SOAS-R-N > 0 was observed in 6/6 patients with excess slow rhythms EEG and 9/11 patients with normal EEG (Odds ratio = 3.4, NS).

In patients with baseline aggressive behavior, a reduction of SOAS-R-S > 3 was observed in 4/6 patients with excess slow rhythms EEG and 4/11 patients with normal EEG (Odds ratio = 3.5, NS).

#### Severity and Frequency of Aggressive Incidents Before Commencing Treatment With Zopiclone

An improvement in CGI-S was observed in 17/22 patients with baseline SOAS-R-N > 1 and 14/23 patients with SOAS-R-N = 0 (Odds ratio 2.2, NS).

#### Zopiclone Treatment Duration and Dose

Zopiclone treatment duration did not predict response. Spearman's coefficient of rank correlation (rho) of zopiclone trial duration and CGI-S, SOAS-R-N and SOAS-R-S change from baseline to LOCF were equal to −0.2, −0.3, and −0.2 respectively, (all NS).

**Figure 1:**
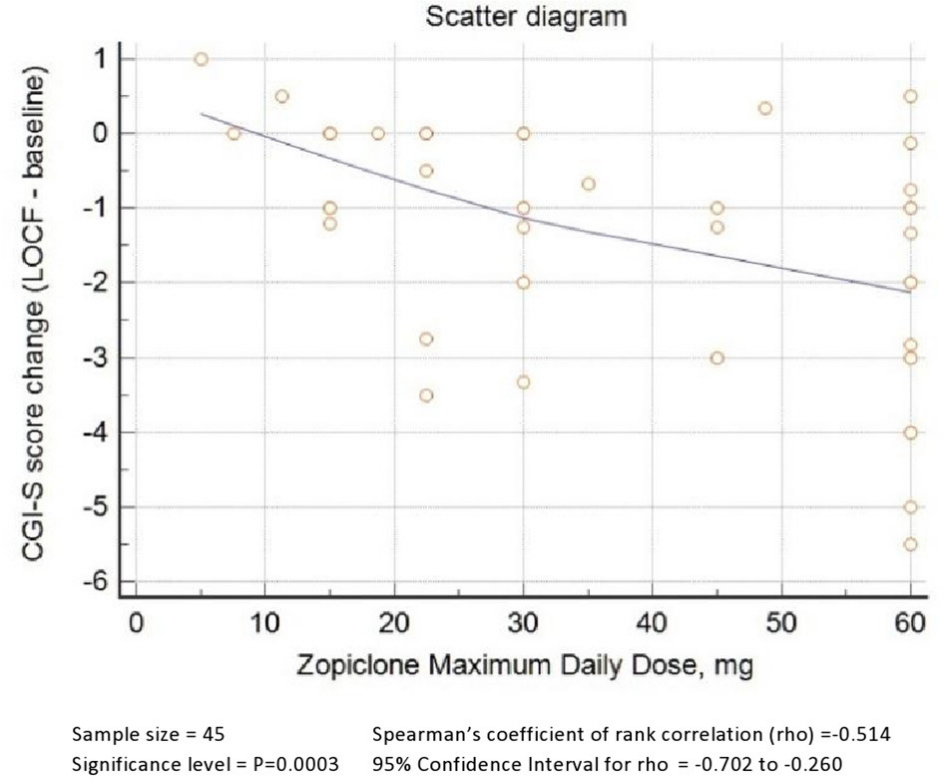
Scatter diagram. Zopiclone maximum daily dose by CGI-S score change.

As shown in Figure 1, zopiclone's maximum daily dose was positively correlated with a better response, as demonstrated by Spearman's rho of zopiclone maximum daily dose and CGI-S change from baseline to LOCF equal to−0.5 (*P* = 0.0003, significant after Bonferroni correction).

**Figure 2:**
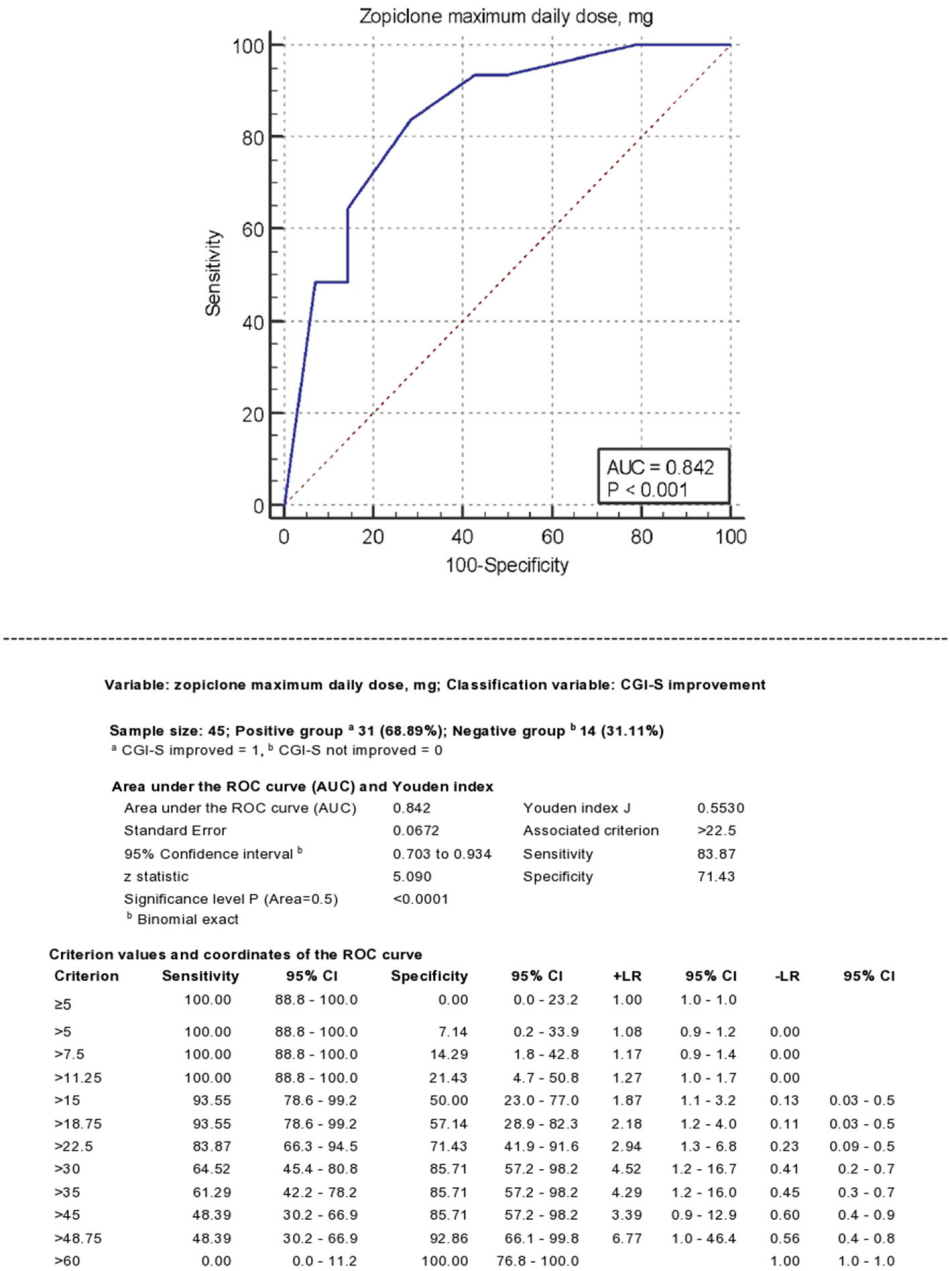
Receiver operating characteristics (ROC) analysis. Classificatory variable: CGI-S improvement.

Figure 2 describes zopiclone's ROC curve maximum daily dose using any reduction of CGI-S score as classificatory variable. The AUC was 0.84 (95% C.I. 0.70–0.93, *P* < 0.0001). A dose of >22.5 mg achieved Youden's index's maximum value, 84% sensitivity and 71% specificity. Doses >30 mg achieved +LR > 4.

Rho of zopiclone maximum daily dose and SOAS-R-N score change (LOCF—baseline) was −0.4 (*P* = 0.0017, significant after Bonferroni correction).

Rho of zopiclone maximum daily dose and SOAS-R-S change from average pre-Z to average post-Z was −0.4 (*P* = 0.0022, significant after Bonferroni correction).

The ROC analysis using SOAS-R-N improvement as classificatory variable provided similar results, with AUC = 0.80 (95% CI 0.58–0.92; *P* = 0.0008) and maximum value of Youden's index achieved at a total daily dose of >30 mg.

The ROC analysis using SOAS-R-S improvement as a classificatory variable could not be performed in patients with baseline aggressive behavior because all patients had a reduction of SOAS-R-S score irrespective of zopiclone dose. The ROC analysis using SOAS-R-S improvement in all 45 patients, with or without baseline aggressive behavior, resulted in AUC = 0.67 (95% CI 0.52–0.81; *P* = 0.0335) and maximum value of Youden's index for the ROC curve achieved at a total daily dose of >30 mg.

## Discussion

In this retrospective chart review, we examined the off-label use of daytime zopiclone in 45 psychiatric inpatients with a baseline median CGI-Severity score of 6 (“severely ill”) and a median LOS of 147 days, which is markedly higher than the median LOS of 20 days recorded in Canada (7). Patients were partially or not responsive to different psychotropic medicines, given alone or in combination and we considered justified the off-label use of high doses of zopiclone only on compassionate grounds, after no other standard treatment could be tried instead. Therefore, these preliminary results cannot be generalized at present to the typical patients with either schizophrenia or mood disorders, who represent the vast majority of psychiatric hospital patients. All patients had a treatment history with benzodiazepines of varying duration without satisfactory benefits. Most patients (82%) were tried on zopiclone because of aggressive behavior and/or a baseline EEG with excess slow theta/delta rhythms, taking in no consideration the underlying psychiatric diagnosis. We hypothesized a favorable response in this type of patients in light of several case reports and studies demonstrating associations between sub-sedative doses of zolpidem (another GABAA PAM) and recoveries from brain damage, stroke, trauma, hypoxia (8) and other neurological disorders (9). In patients with brain damage, zolpidem response has been associated with a shift in EEG power from the delta/theta bands to higher frequency bands (3, 10). The early EEG literature had identified a relationship between excess theta band and aggression in violent offenders (11), a relationship that has been replicated by some authors (12, 13).

Furthermore, there is support for an association between reduced GABA activity and aggression (14). Consistently, benzodiazepines are commonly used to control aggression in psychiatric inpatients, even though their efficacy has been recently questioned (15, 16). In mice, acute treatment with zopiclone produces a marked anti-aggressive effect (17, 18). However, we are not aware of any study of zopiclone to manage aggression in humans.

In our case-series an improvement of illness severity (CGI-S) was observed in 69% of patients, with 60% being much or very much improved. Clinical improvement was observed across different mental state dimensions, including cognition in patients with neurocognitive disorders, motricity, mood, and catatonic features. No effect on positive psychotic symptoms was reported in any of the patients. The dimension most often showing improvement, across this diagnostically mixed group of patients, was aggression. While on daytime zopiclone, 91% of patients with baseline aggression had a clinically relevant reduction in aggressive incidents. Treatment was well-tolerated by most patients, with no severe adverse events. The most common side effects reported in our cohort (sedation, drowsiness, tiredness) are also the most reported side effects of zopiclone's nighttime administration. Insomnia was reported in 2 cases, and this paradoxical side effect has also been rarely observed with nighttime administration (19). Six patients discontinued zopiclone due to non-serious and reversible side effects. Overall, despite using high daytime doses, the tolerability profile was similar to the nighttime administration for insomnia. The excellent tolerability/safety profile observed in this sample is perhaps explained by the fact that zopiclone is widely used to treat insomnia in psychiatric patients. Patients with known intolerance to zopiclone were excluded from its off-label use. Still, doses well-above the maximum recommended were used, and the lack of any severe tolerability issues, including tolerance and tachyphylaxis, is encouraging. We are aware of few patients of this cohort who are still treated in the community with zopiclone (>4 years) and have had a relapse of aggression after tapering attempts. Only two predictors of response obtained statistical significance. Hospital length of stay, predictably, was associated with a worse outcome. Zopiclone's total daily dose was positively correlated with the improvement of both CGI and SOAS-R scores. Zopiclone dose ROC AUCs were statistically significant in predicting the improvement of either CGI or SOAS-R scores. A dose cut-off of >22.5 mg and >30 mg achieved the maximum value of Youden's index in predicting a reduction of CGI-S and SOAS-R-S, respectively. Such a wide range of dosages (5–60 mg) used in this cohort is unusual for any new drug trial. This is likely because there was no guidance available about zopiclone's off-label daytime use and clinicians' diversity (12 psychiatrists in total), as physicians began using it without any formal coordination. Unintentionally, this created the opportunity to assess efficacy at different doses, and the information obtained will prove useful in designing future studies. While efficacy data from open-label studies bear little strength in supporting a drug's efficacy, the statistically significant correlation between the dose of zopiclone and clinical improvement (both global and in a specific outcome measure) represents proof of concept, which encourages further clinical research in this area. It should also be noted that the clinical population described in this paper represented the high end for severity and complexity of all patients admitted to our psychiatric hospital units and such a population would be extremely difficult to enroll in any prospective clinical study. Therefore, our contribution, in spite of its evident methodological shortcomings, may represent the best possible pragmatic approach to investigate the effectiveness of new interventions in complex, difficult-to-treat and violent psychiatric inpatients.

## Study limitations

The selection of patients of this study represents both a strength and a limitation. Studies of new treatments for extremely complex and difficult to treat psychiatric inpatients are rare. At the same time, this study provides no evidence to justify the off-label use of zopiclone in the vast majority of patients admitted to psychiatric wards.

Another important limitation is the design of this study which is not a prospective randomized double-blind comparator-controlled study. However, this study was not hypothesis driven and it was rather inspired by the desire to better understand a new practice in our hospital, which was started after a serendipitous observation.

The inclusion of patients at the peak of severity of their symptoms represents another potential limitation of this study. This is in relation to the spontaneous tendency for patients to improve when they have a maximum of symptoms. This confounder is difficult to quantify in studies without an alternative treatment comparison. However, the correlation between follow-up duration and clinical outcomes was low and not statistically significant (even without Bonferroni correction); which suggests that this phenomenon of spontaneous improvement did not occur in this cohort of difficult to treat patients.

In statistics, the Bonferroni correction of P is a method to counteract the problem of multiple comparisons which increase the chance of observing a rare event, and therefore, the likelihood of incorrectly rejecting a null hypothesis (false positives). We opted for the most conservative method in analyzing the statistical significance of the relationship of multiple demographic and clinical variables to outcomes because of the novelty of our observations and hence the concern of creating false expectations based on a fluke. However, the Bonferroni correction comes at the cost of increasing the probability of producing false negatives, i.e., reducing statistical power. For that reason, new methods of P correction have been proposed (such as the Benjamini-Hochberg adjusted *P*-value). Nevertheless, using the Benjamini-Hochberg method the statistical significance of the 2 variables with uncorrected *P* < 0.05 remained not significant after correction (adjusted *P* = 0.1).

Tobacco smoking is another important confounder in studies of aggression which was not taken in consideration in the study, because information on smoking behavior was not routinely recorded in the charts. Although tobacco smoking is not known to interact with the metabolism of zopiclone, it can still interact with other medications which could have been co-administered. Furthermore, smoking withdrawal is known to increase hostility and aggression. Nevertheless, we believe that the impact of tobacco smoking and tobacco smoking withdrawal was not important in our particular setting. In fact, smoking is forbidden in our PICU and patients in other wards are not allowed to smoke until they are improved enough to obtain unescorted ground privileges. All smokers have almost unlimited access to nicotine replacement.

Contrary to our expectation, a baseline resting EEG showing excess delta/theta waves could not predict clinical response. Nevertheless, the EEG of some patients normalized, in parallel with clinical improvement, while treated with zopiclone. Our negative findings on the predictive value of EEG may be related to having used qualitative reports instead of quantitative analysis, which represents an obvious limitation.

## The Way forward

Assuming that future well-designed studies will confirm the efficacy of zopiclone (and other selective GABA PAMs) in reducing aggression and other clinical dimensions in difficult-to-treat neuropsychiatric patients, the question about the possible mechanism of action deserves some preliminary exploration. We speculate here about 2 potential competing mechanisms of action. The first mechanism would involve the remediation of thalamocortical dysrhythmia, which has been postulated to be a common pathophysiological mechanism of multiple chronic difficult-to-treat neuropsychiatric disorders (20).

Zopiclone is highly selective for α5 subunit, medium binding to α2, α3 but lower toward α1 on GABAA receptor (21). It has been shown that dendrite-targeting somatostatin interneurons and NO-synthase-positive neurogliaform cells preferentially activate α5-subunit- containing GABAA receptors (α5-GABAARs), generating slow inhibitory postsynaptic currents (IPSCs) in hippocampal CA1 pyramidal cells; furthermore the synaptic activation of these receptors can very effectively control voltage-dependent NMDA-receptor activation as well as Schaffer-collateral evoked burst firing in pyramidal cells (22). This discovery reinforces the concept that α5-GABAARs represent promising drug targets for the treatment of several neurological and psychiatric conditions, which may include neurodevelopmental disorders, cognitive impairment in aging, depression, and schizophrenia (23, 24).

An alternative mechanism of action of zopiclone would involve immunomodulatory and anti-inflammatory properties.

Since the early descriptions of anti-NMDA-receptor encephalitis (25–27), the diagnosis of autoimmune encephalitis (AE) is now made more frequently, the majority of cases are not paraneoplastic, involve multiple protein targets of autoantibodies, other than NMDA receptors, and may also present with a relapse-remitting or a chronic course (28, 29). Autoimmunity has long been invoked in the pathophysiology of autism (30). In a recent systematic review, studies provided varying levels of evidence that Autism Spectrum Disorder children displayed higher levels of antibodies reactive to folate receptor α, MAG, MBP, ribosome P, endothelial cell, and ANA, as compared to healthy controls (31). Autoimmunity has also been invoked to explain the pathophysiology of subgroups of patients with schizophrenia spectrum and other psychotic disorders (32, 33), mood disorders (34, 35), PTSD (36–38), neurocognitive disorders (39, 40), including those secondary to traumatic brain injury (41, 42).

The potential therapeutic role of zopiclone in autoimmune psychiatric syndromes could be explained by a non-specific immunomodulatory effect or a specific protection against the neurotoxic effects of autoantibodies for GABAA receptors. In recent years it has been discovered that GABA can modulate the immune response (43–45). Consistently, few case reports suggest that zolpidem can be beneficial in the management of NMDA AE (46, 47). The demonstration of autoantibodies against the GABAA receptor in human sera from patients diagnosed with encephalitis who presented with cognitive impairment and multifocal brain MRI abnormalities (48) opens the possibility that drugs binding to the GABAA receptors could compete with the autoantibodies for binding on the same target and offer protection against autoimmune neurotoxicity. This concept has been proposed by Zimering et al. (42). The authors found that nearly two-thirds of their sample of traumatic brain injured-patients harbored 5-HT2AR autoantibodies in their circulation. Plasma containing these autoantibodies caused neurite retraction in mouse N2A neuroblastoma cells and accelerated N2A cell loss which was subsequently prevented by co-incubation with highly selective 5-5HT2AR antagonists, such as spiperone and ketanserin.

## Data Availability Statement

The original contributions presented in the study are included in the article/Supplementary Material, further inquiries can be directed to the corresponding author/s.

## Ethics Statement

This retrospective study, involving human participants, was reviewed and approved by University of Alberta Research Information Services (Pro00072813). Written informed consent for participation was not required for this study in accordance with the national legislation and the institutional requirements.

## Author Contributions

AC-N and EB: had full access to all of the data in the study and take responsibility for the integrity and the accuracy of the data analysis. AC-N: concept and design and statistical analysis. AC-N, DL, LB, and EB: drafting of the manuscript. AC-N, EB, DL, LB, ZL, and MS: critical revision of the manuscript for important intellectual content. All authors acquisition, analysis, or interpretation of data.

## Conflict of Interest

The authors declare that the research was conducted in the absence of any commercial or financial relationships that could be construed as a potential conflict of interest.

## Publisher's Note

All claims expressed in this article are solely those of the authors and do not necessarily represent those of their affiliated organizations, or those of the publisher, the editors and the reviewers. Any product that may be evaluated in this article, or claim that may be made by its manufacturer, is not guaranteed or endorsed by the publisher.

## Footnotes

^1^Data for the period 2016–2018 were not available.
