# Supplementary figures and images for: Retrospective Observational Study of Daytime Add-On Administration of Zopiclone to Difficult-to-Treat Psychiatric Inpatients With Unpredictable Aggressive Behavior, With or Without EEG Dysrhythmia
Source: Front Psychiatry. 2021 Aug 17;12:693788. doi: 10.3389/fpsyt.2021.693788 (PMC8415882; doi:10.3389/fpsyt.2021.693788)

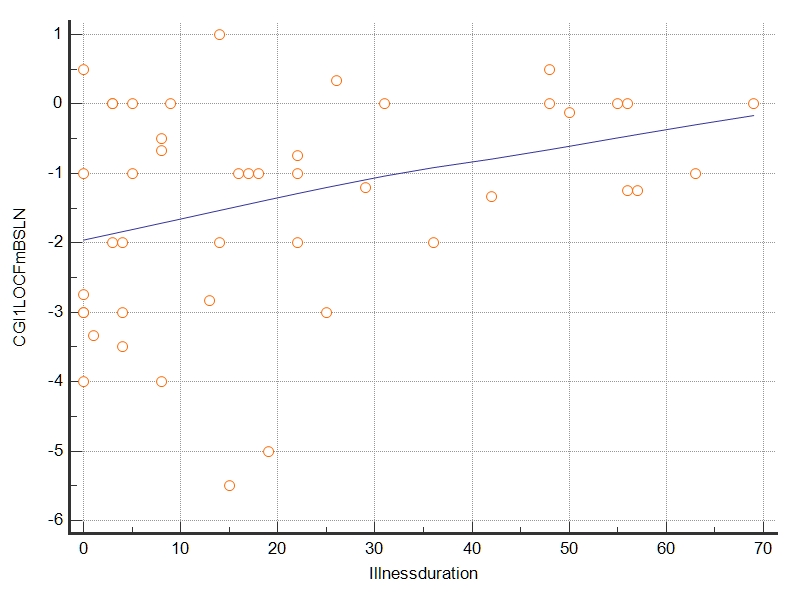

Supplement: Supplementary file 1 [file Image_1.JPEG]
